# Eight Years With an Airway Foreign Body: Asthma or Aspiration?

**DOI:** 10.7759/cureus.104568

**Published:** 2026-03-02

**Authors:** Nicole Henry, Hubert A Benzon, Kathleen L Boyne, Ashley Sarver, Laura Rosenthal, Faith Svigos, Veronica Drozdowski, Jonathan Shaffer, Michael A Evans

**Affiliations:** 1 Pediatric Anesthesiology, Northwestern University Feinberg School of Medicine, Chicago, USA; 2 Pulmonary Medicine, Northwestern University Feinberg School of Medicine, Chicago, USA; 3 Otorhinolaryngology-Head and Neck Surgery, Northwestern University Feinberg School of Medicine, Chicago, USA

**Keywords:** airway foreign body, aspiration, asthma, foreign body aspiration, pediatric anesthesiology

## Abstract

Foreign body aspiration (FBA) is most commonly observed in children under the age of three and typically presents acutely with respiratory distress. In adolescents, FBA is less frequent and often results from behavioral incidents or accidental inhalation. Chronic retained airway foreign bodies are rare and often present with nonspecific or misleading symptoms, making timely diagnosis challenging.

This case highlights the unusual presentation and anesthetic considerations in managing a delayed diagnosis of FBA in a 15-year-old male with a history of mild intermittent asthma who presented with new-onset hemoptysis, hematemesis, and a chronic, malodorous cough for a duration of eight months. Symptoms were followed by a coughing episode that produced blood-streaked sputum and post-tussive emesis. Imaging revealed left lower lobe bronchiectasis and mucus-impacted airways. Notably, at age seven, the patient choked on a pen cap and sought medical attention but was discharged after a negative nasolaryngoscopy and was instructed to observe for passage of the pen cap in their stool.

Bronchoscopy was planned, given the patient's history, presentation, and new CT findings. Under anesthesia with preserved spontaneous ventilation via a supraglottic airway, flexible bronchoscopy identified a pen cap lodged in the distal left lower lobe. Subsequent rigid bronchoscopy was performed due to the depth of the object and associated airway changes. The foreign body was successfully retrieved, and postoperatively, the patient’s respiratory symptoms markedly improved. They recovered after a course of antibiotics and airway clearance therapy.

This case underscores the importance of maintaining clinical suspicion for FBA in patients with chronic respiratory symptoms with relevant history, regardless of time elapsed. It also highlights key anesthetic considerations in managing airway access, ventilation, and procedural coordination during rigid bronchoscopy. The successful outcome illustrates the necessity of interdisciplinary collaboration and individualized anesthetic planning in complex airway foreign body retrieval.

## Introduction

Foreign body aspiration (FBA) is a well-recognized airway emergency, most commonly observed in children under the age of three [1-3]. However, it can also occur in adolescents, often due to behavioral factors or accidental inhalation. Recognition of an aspirated airway foreign body is typically immediate, as it frequently presents with dramatic symptoms such as coughing, choking, or acute respiratory distress. Even when initial symptoms are subtle, most cases are identified early due to persistent respiratory findings. It is therefore unusual for a patient to unknowingly live with a foreign body in their airway for an extended period of time.

The diagnosis and management of FBA rely heavily on clinical suspicion, imaging, and ultimately, bronchoscopic evaluation. Rigid bronchoscopy remains the standard approach for both diagnosis and removal; thus, the anesthetic plan must be carefully tailored to the location of the object, the patient’s respiratory status, and the procedural requirements [4,5]. Anesthetic management presents unique challenges, particularly in preserving oxygenation while maintaining access to a compromised airway.

We present herein the case of a 15-year-old patient with a prolonged history of unexplained respiratory symptoms, ultimately found to have a pen cap lodged in the distal airway. This case highlights the atypical presentation of chronic FBA and the anesthetic considerations essential to successful retrieval. Written Health Insurance Portability and Accountability Act (HIPAA) authorization/consent for publication of the present case was obtained from the patient’s guardian, and this article adheres to the applicable Enhancing the QUAlity and Transparency of health Research (EQUATOR) guidelines.

## Case presentation

**Figure 1:**
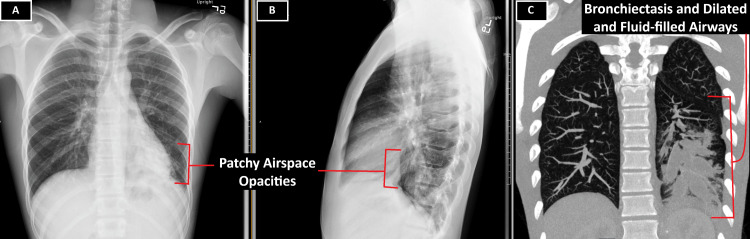
Chest X-ray and CT chest with contrast A and B: The patient's AP and lateral chest X-rays revealed patchy airspace opacities within the left lower lobe, thought to represent pneumonia. C: The patient's CT chest with contrast showed bronchiectasis and dilated and fluid-filled airways can be visualized throughout the left lower lobe.

A 15-year-old male with a history of mild intermittent asthma and migraines presented to the emergency department with new-onset hemoptysis and hematemesis in the setting of a chronic cough for eight months. A coughing fit led to the expectoration of one teaspoon of blood, followed by post-tussive emesis also containing blood. After the episode, the patient had new-onset, sharp, left-sided back pain that worsened with inspiration. The patient also presented with new-onset dyspnea. Further history revealed a now-malodorous chronic cough and that the patient had been diagnosed with imaging-verified left lower lobe pneumonia at another hospital one week prior (Figure 1, panels A-B). The pneumonia was treated with amoxicillin-clavulanate for seven days, along with four days of prednisone and albuterol two times a day. Additionally, when asked about prior medical history and hospitalizations, the patient and family reported a hospital visit eight years ago (at the age of seven) after choking on a pen cap. At that time, flexible nasolaryngoscopy was performed, which was unrevealing. The patient was discharged after this nasolaryngoscopic evaluation and instructed to monitor their stool for passage of the pen cap. During that hospital visit, no cross-sectional imaging was performed. After admission to our hospital, a CT scan of the chest with contrast (Figure 1, panel C) revealed left lower lobe bronchiectasis, dilated fluid/mucus-impacted airways, and ground-glass airway opacification.

Differential diagnosis, investigations, and treatment

After consultation with the pulmonology service, the decision was made to proceed to the operating theater to assess pulmonary pathology with bronchoscopy, given the differential diagnosis included infectious diseases (such as tuberculosis or fungi) or an airway FBA from the distant history of choking on a pen cap. Anesthesia was induced intravenously with lidocaine, fentanyl, and propofol, and a supraglottic airway was placed to maintain spontaneous ventilation. Anesthetic depth was initially maintained with sevoflurane but was transitioned to a combination of inhaled sevoflurane and propofol infusion, given the use of the intermittent surgical insufflation technique and the patient’s high anesthetic tolerance.

Flexible bronchoscopy was performed by the pulmonology team using the supraglottic airway as a conduit, with the patient breathing spontaneously. Their flexible bronchoscopy revealed a retained foreign body in the left lower lobe consistent with a chewed-up pen cap. A large amount of purulent, blood-tinged secretions was present throughout the left lung. There were also several areas of focal narrowing along the airway lumen, with post-stenotic dilation distally. As the foreign body could not be retrieved with a flexible bronchoscope, airway instrumentation transitioned to the otolaryngology team for rigid bronchoscopy.

**Figure 2:**
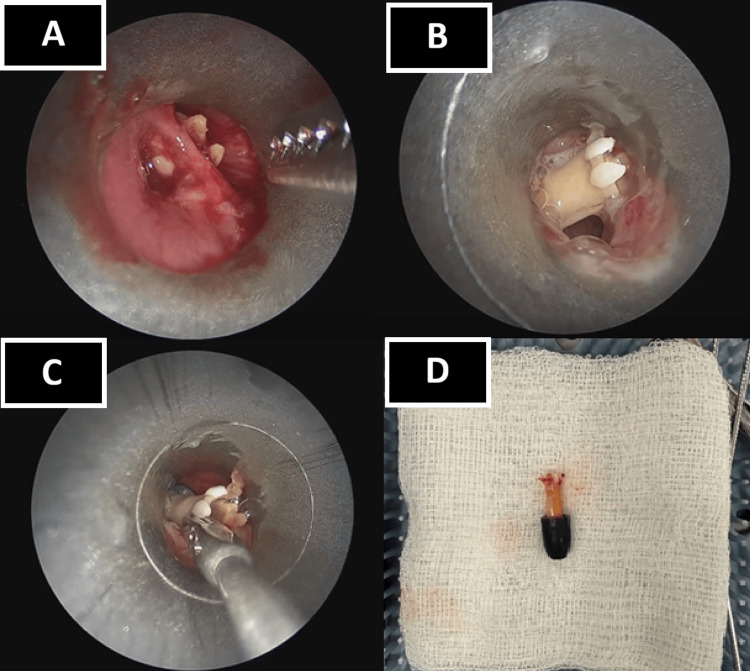
Intraoperative images of the airway foreign body A: The pen cap stuck behind a scar band in a narrowed distal airway; B: The pen cap (now sideways in the airway) after lysis of the scar band and before extraction; C: The pen cap being grasped and extracted; D: Extracted airway foreign body (the chewed pen cap)

To facilitate rigid bronchoscopy, the supraglottic airway was withdrawn, direct laryngoscopy with a BritePro Solo Phillips Blade, #2 laryngoscope (Flexicare, Mountain Ash, Wales, UK) was performed, and an adult-size 6.5 rigid bronchoscope was passed distally to the level of the foreign body. During this part of the procedure, the patient continued to breathe spontaneously, with constant insufflation of fresh gas via the bronchoscope, and did not experience desaturation events. Anesthesia was maintained with propofol infusion with supplemental bolus doses of propofol throughout the rigid bronchoscopy. To pass the scope far enough and mobilize the foreign body, two scar bands in the left mainstem bronchus were lysed sharply using optical scissors (Figure 2, panels A-C; Video 1). Minor bleeding encountered during lysis was managed with direct suctioning through the rigid bronchoscope and brief pauses to allow visualization and spontaneous hemostasis. No topical vasoconstrictors or cautery were required, and oxygenation remained stable throughout the procedure. The foreign body was grasped with a forceps, extracted, and confirmed to be a pen cap (Figure 2, panel D; Video 2).

**Video 1:** A video recording from the patient’s rigid bronchoscopy During the extraction of the airway foreign body, a scar band that trapped the pen cap was encountered and had to be lysed prior to successful removal.

**Video 2:** Video recording from the patient’s rigid bronchoscopy showing the successful removal of the foreign body

The patient was emerged from anesthesia and taken to the postanesthesia care unit. There, they were weaned from supplemental oxygen and transferred to the postsurgical floor, with immediate relief of their presenting respiratory symptoms. Given the presence of an airway foreign body, the patient transitioned to ampicillin-sulbactam for antibiotic coverage. They were also initiated on vest physiotherapy, the Portex acapella DH vibratory positive expiratory pressure (PEP) system with mouthpiece device (ICU Medical Inc., San Clemente, CA, USA), and inhaled 3% hypertonic saline. The patient was prescribed albuterol for enhanced pulmonary clearance, given the chronic respiratory symptoms and CT-confirmed bronchiectasis. Intraoperative cultures grew amoxicillin-clavulanate-sensitive organisms, so the patient was discharged on oral antibiotics.

## Discussion

This 15-year-old male with a history of mild intermittent asthma who presented with chronic progressive cough and hemoptysis was ultimately found to have bronchiectasis and a chronic retained airway foreign body. This episode was the first time their chronic coughing resulted in blood, despite the pen cap having been aspirated eight years prior. Additionally, their asthma diagnosis occurred eight months before the hemoptysis episode.

Asthma is the most common chronic respiratory disease of childhood. It is often associated with airway hyperresponsiveness to stimuli and chronic airway inflammation. Distinguishing other pediatric respiratory conditions from asthma can be challenging in children due to the variability in clinical presentations and overlapping symptoms. The Global Initiative for Asthma (GINA) guidelines recommend the diagnosis of asthma in children be based on a combination of the following: 1) pattern of recurrent symptoms (cough, wheeze, shortness of breath, or chest tightness) that vary in intensity over time due to triggers such as exercise, allergen exposure, or viral illnesses; 2) variable expiratory airflow limitation in those able to perform spirometry; 3) documented response to asthma therapy (i.e., response to inhaled corticosteroids); and 4) exclusion of alternate diagnoses [6].

This patient did have some typical features and risk factors, including a history of prematurity, a strong family history of asthma, and typical triggers of respiratory symptoms (exercise, viruses) with a reported response to bronchodilators. However, there are features highlighted in the GINA guidelines that decrease the probability that respiratory symptoms are attributable to asthma, including chronic sputum production as experienced by our patient over the preceding eight months. Although the retained foreign body was unilateral, localized airway obstruction can produce symptoms that clinically resemble asthma, including cough, exertional dyspnea, and intermittent wheeze. Airflow limitation from partial obstruction may generate a monophonic wheeze that is misinterpreted as diffuse bronchospasm, particularly when spirometry is not performed or when response to bronchodilators is subjective. Additionally, chronic airway inflammation distal to the obstruction may lead to secondary airway hyperresponsiveness, further mimicking asthma physiology. Prior case reports have documented endobronchial foreign bodies initially misdiagnosed as asthma for similar reasons [7,8]. This history further supported the decision to challenge the underlying asthma diagnosis and proceed with additional evaluation, including bronchoscopy to assess for possible retained foreign body.

Foreign body aspiration is most frequently encountered in the pediatric population, particularly among children under the age of three [1-3]. The diagnosis is typically made shortly after the aspiration event, prompted by symptoms such as sudden coughing, choking, or respiratory distress. In contrast, chronic aspiration with prolonged retention of a foreign body is uncommon, especially in older children and adolescents, and often presents with nonspecific or evolving respiratory symptoms. At our quaternary pediatric referral center, airway FBA remains a regularly encountered indication for rigid bronchoscopy, with most cases presenting acutely rather than in a delayed fashion. This case of a 15-year-old patient with a retained pen cap demonstrates an unusual presentation of FBA, in which the foreign body remained undiagnosed for eight years.

The prolonged asymptomatic interval following aspiration likely reflects several pathophysiologic factors. Distally lodged foreign bodies may not cause complete airway obstruction, allowing partial ventilation and gradual accommodation of airflow around the object. Inert materials such as plastic may initially provoke minimal mucosal inflammation, permitting a clinically silent period before progressive granulation tissue formation, airway remodeling, and secondary infection develop. Over time, chronic low-grade obstruction can result in mucus retention, recurrent infection, and localized bronchiectasis, eventually leading to more overt symptoms such as malodorous sputum or hemoptysis. Similar delayed presentations have been reported in cases of chronic retained foreign bodies lasting years to decades before diagnosis [7-9]. 

The initial failure to identify and retrieve the aspirated pen cap, despite a history of choking and a missing object, may have stemmed from reliance on flexible nasolaryngoscopy, which lacks the sensitivity to detect foreign bodies located in the distal airways. Failure to perform chest imaging or bronchoscopy likely contributed to the missed diagnosis. This highlights the critical diagnostic pitfall in pediatric FBA of sole reliance on insufficient airway evaluation modalities in the acute setting [10,11]. As the patient aged, the retained foreign body likely contributed to chronic airway inflammation, infection, and ultimately bronchiectasis, as evidenced by CT findings. These changes reflect the well-documented progression of tissue injury associated with long-standing airway obstruction and infection. Delayed diagnosis can lead to significant pulmonary complications, including chronic infection, airway obstruction, and irreversible parenchymal changes such as bronchiectasis. Chronic FBA may present months to years after the initial event with nonspecific findings such as chronic cough, recurrent pneumonia, unilateral wheeze, hemoptysis, or bronchiectasis, often leading to misdiagnosis as asthma or persistent infection [6-11]. In this patient, chest CT demonstrated left lower lobe bronchiectasis, mucus plugging, and post-obstructive changes, all consistent with prolonged airway inflammation due to chronic foreign body retention.

From an anesthetic standpoint, airway management in such cases is complex. The primary objectives include maintaining adequate anesthesia alongside oxygenation, minimizing airway trauma, and allowing procedural access for bronchoscopy. In this case, a supraglottic airway was used to preserve spontaneous ventilation during initial flexible bronchoscopy. When rigid bronchoscopy became necessary, careful coordination between the anesthesia and otolaryngology teams allowed for safe removal of the foreign body, despite the patient’s high anesthetic tolerance, distal airway involvement, and requisite lysis of adhesions proximal to the foreign body to facilitate removal. Given the need for lysis of adhesions to access and retrieve the foreign body, the use of a multidisciplinary team, including anesthesiology, pulmonology, and otolaryngology, was critical to the successful management of this case.

Anesthetic management of chronic FBA differs in important ways from acute aspiration events. In acute FBA, the primary concern is complete airway obstruction with risk of sudden hypoxia or dislodgement of the object during positive pressure ventilation, often necessitating strict maintenance of spontaneous ventilation and rapid rigid bronchoscopy [5]. In contrast, chronic foreign bodies are more commonly associated with distal obstruction, airway inflammation, granulation tissue, bronchiectasis, and friable mucosa. These changes increase the risk of bleeding, airway edema, and difficulty with extraction due to adhesions or scar bands, as encountered in this case. Patients may also have reactive airway disease from chronic inflammation, increasing the risk of bronchospasm during airway manipulation. Therefore, anesthetic goals in chronic FBA include careful titration of depth to minimize coughing and bronchospasm, preparedness for bleeding and impaired visualization, and anticipation of longer procedural time due to tissue remodeling. Multidisciplinary coordination becomes particularly important when airway scar lysis or complex retrieval is required.

## Conclusions

This case underscores the need for high clinical suspicion of FBA in pediatric and adolescent patients with chronic respiratory symptoms, particularly when there is a known history of aspiration. It also illustrates the long-term pulmonary consequences of missed FBA and emphasizes the value of multidisciplinary collaboration in both diagnosis and management. Early use of appropriate imaging and bronchoscopic evaluation is critical in avoiding delayed diagnosis and irreversible pulmonary damage.
